# Cellulose Nano-Films as Bio-Interfaces

**DOI:** 10.3389/fchem.2019.00535

**Published:** 2019-07-30

**Authors:** Vikram Singh Raghuwanshi, Gil Garnier

**Affiliations:** Bioresource Processing Research Institute of Australia (BioPRIA), Monash University, Clayton, VIC, Australia

**Keywords:** cellulose, thin film, biomolecule, interface, diagnostics, characterization

## Abstract

Cellulose, the most abundant polymer on earth, has enormous potential in developing bio-friendly, and sustainable technological products. In particular, cellulose films of nanoscale thickness (1–100 nm) are transparent, smooth (roughness <1 nm), and provide a large surface area interface for biomolecules immobilization and interactions. These attractive film properties create many possibilities for both fundamental studies and applications, especially in the biomedical field. The three liable—OH groups on the monomeric unit of the cellulose chain provide schemes to chemically modify the cellulose interface and engineer its properties. Here, the cellulose thin film serves as a substrate for biomolecules interactions and acts as a support for bio-diagnostics. This review focuses on the challenges and opportunities provided by engineering cellulose thin films for controlling biomolecules interactions. The first part reviews the methods for preparing cellulose thin films. These are by dispersing or dissolving pure cellulose or cellulose derivatives in a solvent to coat a substrate using the spin coating, Langmuir-Blodgett, or Langmuir-Schaefer method. It is shown how different cellulose sources, preparation, and coating methods and substrate surface pre-treatment affect the film thickness, roughness, morphology, crystallinity, swelling in water, and homogeneity. The second part analyses the bio-macromolecules interactions with the cellulose thin film interfaces. Biomolecules, such as antibodies and enzymes, are adsorbed at the cellulose-liquid interface, and analyzed dry and wet. This highlights the effect of film surface morphology, thickness, crystallinity, water intake capacity, and surface pre-treatment on biomolecule adsorption, conformation, coverage, longevity, and activity. Advance characterization of cellulose thin film interface morphology and adsorbed biomolecules interactions are next reviewed. X-ray and neutron scattering/reflectivity combined with atomic force microscopy (AFM), quartz crystal microbalance (QCM), microscopy, and ellipsometer allow visualizing, and quantifying the structural morphology of cellulose-biomolecule interphase and the respective biomolecules conformations, kinetics, and sorption mechanisms. This review provides a novel insight on the advantages and challenges of engineering cellulose thin films for biomedical applications. This is to foster the exploration at the molecular level of the interaction mechanisms between a cellulose interface and adsorbed biomolecules with respect to adsorbed molecules morphology, surface coverage, and quantity. This knowledge is to engineer a novel generation of efficient and functional biomedical devices.

## Introduction

This review presents the methods and characterization techniques to engineer and quantify the properties of thin and smooth cellulose nanofilms. These model bio-interfaces aim to characterize the bio-molecule morphology and interaction with cellulose for biomedical applications, especially as diagnostics.

Cellulose is the most abundant polymer on the planet with plants producing 180 billion ton per year by photosynthesis from the conversion of CO_2_ and water. Cellulose has widely been used for 2 centuries in commodity and industrial products such as pulp and paper, textile, and coatings. Available on all the inhabited continents, cellulose is biodegradable, non-toxic, sustainable, easy to functionalize, wettable, and flexible (Atalla and Vanderhart, 1984; Habibi et al., 2010b). These properties make cellulose an attractive material for engineering advanced applications such as low-cost, biodegradable, and disposable biodiagnostics (Su et al., 2012; Arcot et al., 2015), point of care analytical devices (Cranston et al., 2010), and membranes (Sukma and Culfaz-Emecen, 2018) for separation (dialysis). Currently, the main biomedical applications of cellulose include biodiagnostics, such as blood typing (Guan et al., 2014; Li et al., 2014; Casals-Terre et al., 2018), and pregnancy tests (Halpern et al., 2008; Koczula and Gallotta, 2016), tissue engineering (Courtenay et al., 2018; Torgbo and Sukyai, 2018; Curvello et al., 2019), scaffolds (Novotna et al., 2013; Modulevsky et al., 2014; Demitri et al., 2016; Courtenay et al., 2017; O'donnell et al., 2018), eye care solutions (Vehige et al., 2003; Luchs, 2010; Nguyen and Latkany, 2011; Racic et al., 2019), coatings (Jimenez et al., 2017; Spera et al., 2017; Sharif Hossain et al., 2018), packaging (Czaja et al., 2007; Sunasee et al., 2016; Shaghaleh et al., 2018), and sensors (Zhu et al., 2014; Ummartyotin and Manuspiya, 2015; Chen et al., 2018).

Cellulose is a hierarchical material. Cellulose fibers from plants are made up of bundles regrouping smaller units of linear monomer chains (Dufresne, 2013). The chain consists of both crystalline and amorphous regions. The crystalline/amorphous phase ratio determines the water intake capacity of the cellulose structure (Kontturi et al., 2011a, 2013). Each cellulose monomeric unit has 3 liable -OH groups which allow cellulose interfaces to be functionalized easily.

The cellulose fiber bundles can be fibrillated to fibrils of nanoscale thickness and length (1–100 nm) by mechanical (Bhatnagar and Sain, 2005; Bandera et al., 2014; Menon et al., 2017), enzymatic (Hu et al., 2018; Narkpiban et al., 2019), or chemical treatment (Sacui et al., 2014; Syafri et al., 2018; Tibolla et al., 2018). The resulting cellulose nanofibers (CNF) are 20–100 nm thick and 1–2 μm long, while the cellulose nanocrystals (CNC) are 5–10 nm in diameter with length ranging between 30 and 100 nm (Xu et al., 2013; Kim et al., 2015; Blanco et al., 2018). Engineered materials from CNF and CNC show high mechanical strength, optical transparency, and thermal stability compared to their original bulk counterparts. Therefore, CNF and CNC are used to produce transparent sheets, hydrogels, and as nanoscale thin film coatings.

Most disposable bio-diagnostics are currently made of polyolefin materials. Their major drawbacks are not being biodegradable which leads to accumulation and environmental toxicity, poor wettability, and difficulty to functionalize. In the last decade, cellulose based papers have been used as substrate in bio-diagnostic to identify pathogens, and to quantify biomolecules interactions (Then and Garnier, 2013). Cellulose based bio-diagnostics are typically used as dipstick (Free et al., 1957; Hosseini et al., 2017), or microfluidics (Martinez et al., 2010; Uddin et al., 2019) to interact with biomolecules. In particular, cellulose paper for blood typing takes half a minute to provide direct evidence, and visual readout outcome of blood groups (Then et al., 2015). In a typical reaction, the blood antigens interact with the pre-adsorbed biomolecules at the cellulose paper interface.

The main challenge associated with using cellulose based paper bio-diagnostics is engineering functionalized surface interacting with the targeted biomolecule for a specific biomedical application. Another issue is to maintain biomolecules activity efficiency over time by preventing their premature aging upon adsorption and immobilization on a surface. Other drawbacks of paper are controlling the fiber mesh network, pore size, and fiber strength. These variables significantly affect diffusion and biomolecules loss in the fiber network, and its activity (Derikvand et al., 2016; Huang et al., 2017; Prathapan et al., 2018). Such challenges can be overcome by engineering cellulose nanoscale thin film as substrate for biomolecules immobilization.

**Figure 1:**
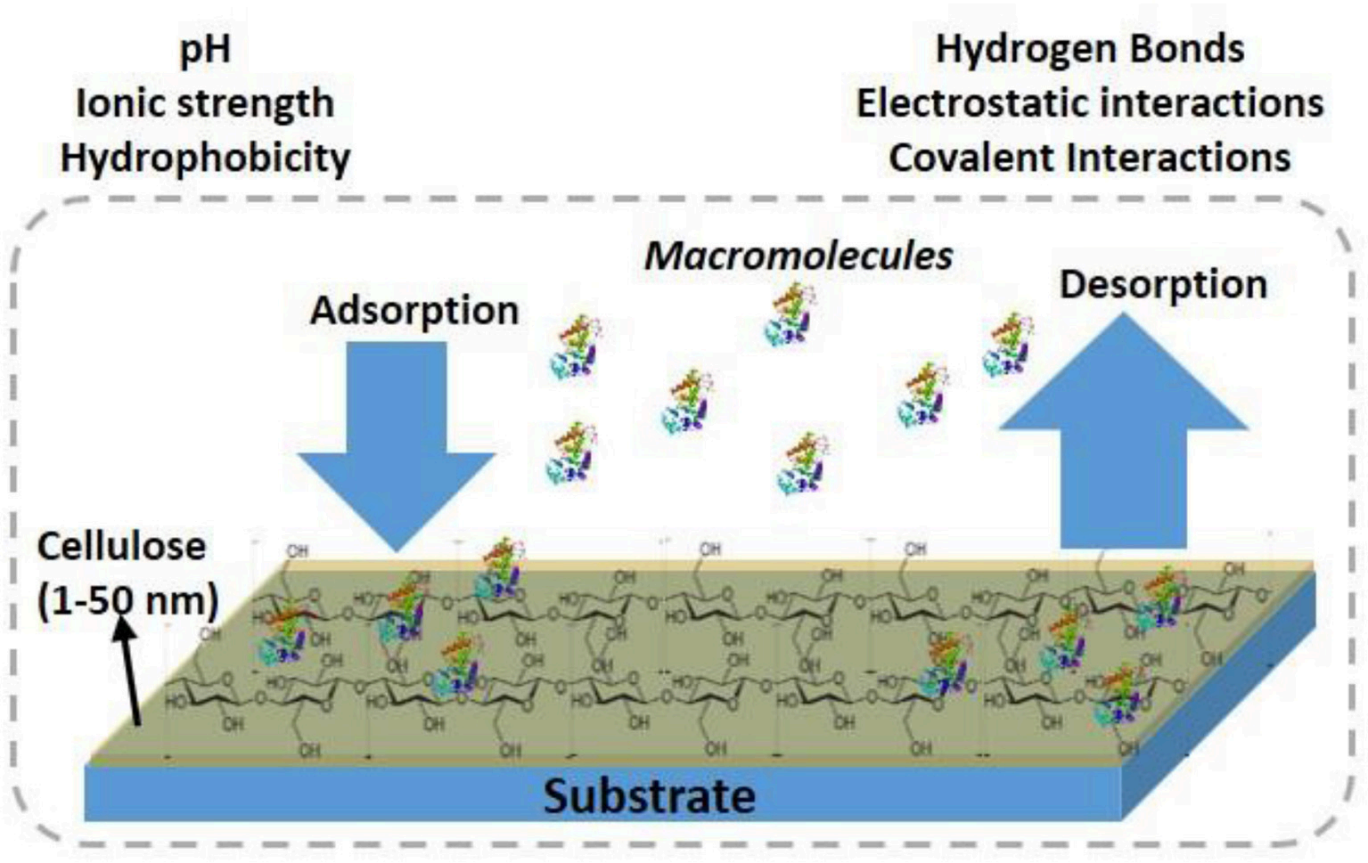
Schematic of antibodies adsorption/desorption at the cellulosic thin film interface.

Cellulose thin films 5–100 nm thick with a roughness <3 nm provide a large surface area for biomolecules to interact, and for interface functionalization. This large available surface allows biomolecules to interact, adsorb, and remain immobilized at high density, and hopefully under controlled morphology onto the cellulose interface (Figure 1).

Cellulose thin films can be prepared with cellulose fibers (CNF, CNC) from plants, and bacteria or by using cellulose derivatives. Different cellulose sources, preparation procedures and coating methods result in film of different morphologies, and crystallinity. Kontturi et al. (2006) reviewed a detailed procedure to prepare cellulose thin film. The formed cellulose film adhere directly or indirectly (pre modified surface) with the substrate by forming electrostatic, van der Waal or hydrophobic forces, and covalent bonds (Eriksson et al., 2007).

The properties of cellulose thin film interface can be optimize by: (i) functionalization of the interface by taking advantage of the 3 liable -OH groups per cellulose monomer, (ii) controlling the ratio of crystalline and amorphous phase (iii) controlling CNF/CNC assembly/organization using magnetic field (Sugiyama et al., 1992; Revol et al., 1994), shear alignment (Yoshiharu et al., 1997; Ebeling et al., 1999), and electric field alignment (Bordel et al., 2006), (iv) engineering different surface morphology, thickness, roughness, and topography.

The interface functionalization leads to specific binding of targeted biomolecules. The controlled crystalline/amorphous phase ratio provides different film swelling behavior which is useful to optimize the liquid content in films (Aulin et al., 2009). The advantage of anisotropically ordered cellulose fiber thin film or anisotropic film surface morphology is the directional distribution and functionality of the adsorbed biomolecules (Prathapan et al., 2018).

This review focuses on the preparation, characterization and the biomedical applications of cellulose thin film interfaces. It provides insight on the best procedures for the consistent preparation of cellulose thin film of controlled structure and morphology. Further it highlights on optimization of film interface properties by surface functionalization. We hope this review will provide the foundation to researchers from academics and industries for developing better biodiagnostics, sensors, coatings, and substrates.

## Cellulose Film Formation

There are two general approaches used to prepare thin cellulose films; these rely on coating a dissolved cellulose derivative solution or a dispersed suspension of cellulose nanofibers (CNF), or cellulose nanocrystal (CNC) onto a surface. The prepared suspension is coated directly onto an interface or indirectly onto a chemically pre-modified substrate. The cellulose derivative coating requires regeneration into pure cellulose by chemical cleaving and substituting the derivative groups for hydroxyls. Dispersed or modified CNF/CNC coatings do not require a post treatment chemical conversion into cellulose. A chemically pre-modified substrate interface is used to control the adhesion and surface coverage of cellulose film by forming electrostatic, hydrogen or covalent bonds.

Different coating methods exist to cast smooth and uniform cellulose thin films (5–100 nm) on a substrate. These methods include spin coating (SC) (Emslie et al., 1958; Larson and Rehg, 1997; Kontturi et al., 2007), Langmuir-Schaefer (LS) (Aulin et al., 2009; Basavaraja et al., 2011), and Langmuir Blodgett (LB) (Sakakibara et al., 2006; Cohen-Atiya et al., 2007). In SC, a cellulose derivative or a cellulose suspension is applied over a flat substrate followed by substrate spinning at a controlled velocity. This produces smooth, homogenous thin films rapidly (in less than a minute). However, SC requires a flat solid substrate. A large amount of material is wasted during spinning, which needs a fast evaporating organic solvent to produce a good quality film. The solvent evaporation rate and the suspension spreading over the substrate during spinning define the film thickness and its homogeneity.

**Figure 2:**
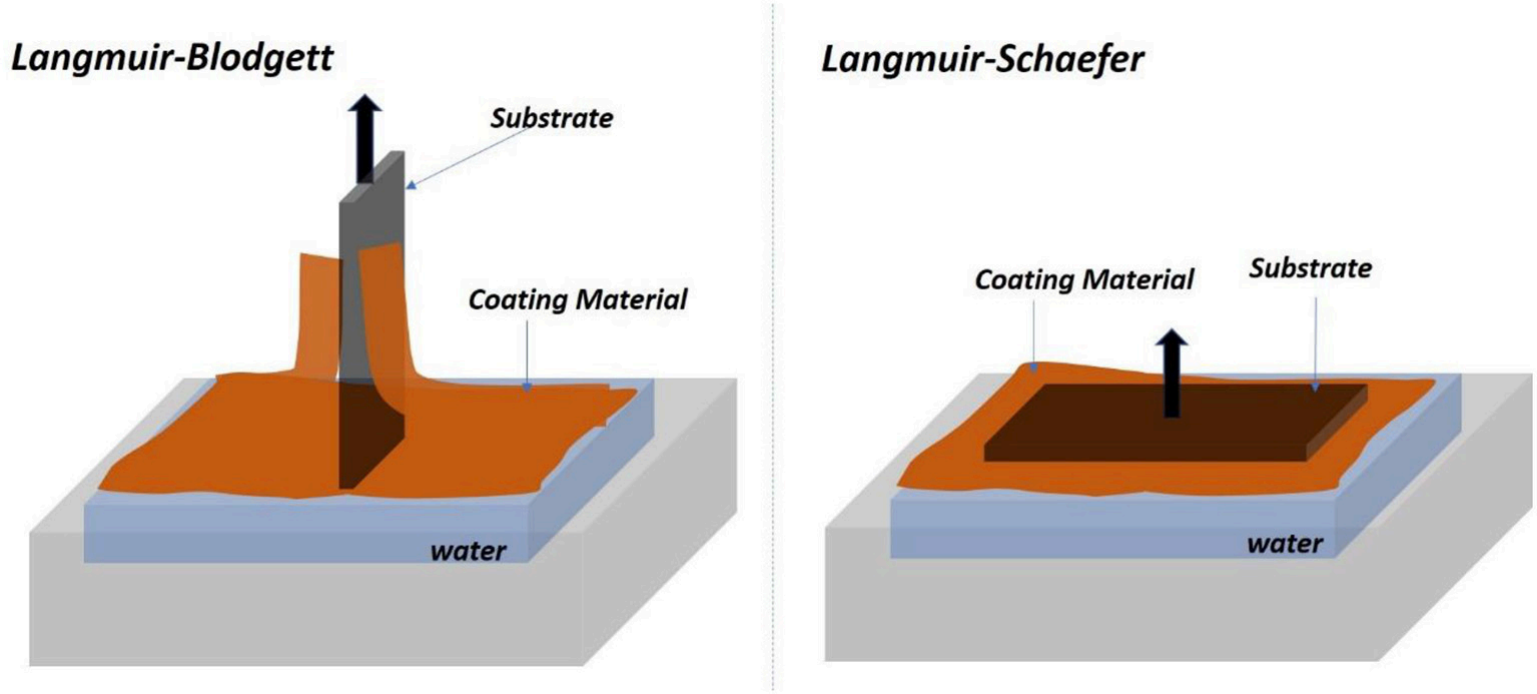
Schematic of the Langmuir-Blodgett **(Left)** and Langmuir-Schaefer **(Right)** set of forming film from the air/water interface.

In LB and LS methods, a cellulose monolayer phase is first formed at the liquid/air interface. The resulting cellulose monolayer is either transferred to a vertically submerged solid substrate forming LB films or onto a horizontally emerged substrate forming LS films, as shown in Figure 2 (Rubinger et al., 2006). However, the deposited films are slow to fabricate and require quick drying in a clean room to prevent impurities deposition. The LB and LS methods have produced nice cellulose films used in many classical studies but they retain some challenging inherent issues.

While cellulose films present many advantages, there are challenges in depositing, and characterizing the cellulose film interface. A first challenge is the dissolution of cellulose or cellulose derivatives in a proper solvent to easily coat a substrate. Acetone and toluene are good volatile solvents for cellulosic solutions, as they evaporate sufficiently fast to produce smooth, and homogeneous films. Cellulose can also be dissolved homogeneously in ionic liquids (IL) but their low vapor pressure hinder coating of smooth and homogenous films (Raghuwanshi et al., 2018). The pure cellulose regeneration process using acid hydrolysis for removing the unwanted derivatives also affect film quality by increasing roughness.

A second challenge in the cellulose film preparation is the formation of cellulose aggregates over the substrate which produces rough and heterogeneous distribution of cellulose at the interface. A homogenous cellulose suspension with a fast drying process are required to hinder the formation of cellulose fibers or crystal aggregates.

Another issue is the chemical modification of CNF, CNC, or the substrate surface is often required for adhering cellulose chains at high surface coverage. The chemical modification might bring toxicity, and add impurities at the interface, affecting surface properties, and biomolecules interaction with cellulose. The interaction between cellulose and modifier polymer depends on the polymer type, functional groups, charge density, and molecular weight (Wagberg, 2000; Ahola et al., 2008).

### Films From Cellulose Derivatives

Trimethylsilyl cellulose (TMSC) is a preferred cellulose derivative for cellulose thin films (Klebe and Finkbeiner, 1969; Cooper et al., 1981; Heinze et al., 2018). Dissolving TMSC in toluene and coating on a substrate (silicon, gold coated) results in thin and homogeneous TMSC films. Exposing the TMSC film surface to HCl acid vapor for 10–15 min substitutes the trimethylsilyl (TMS) groups into hydroxyl groups, regenerating a pure cellulose film (Mohan et al., 2014).

The degree of substitution (DS) is defined as the average number of hydroxyl group per anydroglucosidic unit (3) that has been chemically reacted. It determines the extent of chemical functionalization for cellulose derivatives (Samaranayake and Glasser, 1993). Full derivatization of cellulose into TMSC with a DS 3 is an easy reaction to proceed and to control, which results in a derivative fully soluble into tetrahydrofuran (THF). By contrast, cellulose acetate of DS = 2 is required for full solubility into acetone; interrupting the reaction midway or hydrolyzing cellulose triacetate back to cellulose diacetate (DS = 2) are reactions more challenging to control.

The neutron reflectivity and AFM measurements of the TMSC based cellulose films indicate the formation of a 20 nm thick film with a roughness of 0.5 nm. The measured volume fraction of cellulose in the film is about 80% (Kontturi et al., 2011b). Grazing incidence X-ray diffraction (GI-XRD) measurement shows the film to be amorphous with little traces of cellulose II and III crystallites. The film thickness, roughness, and crystallinity remain unchanged during the swelling and drying process.

**Figure 3:**
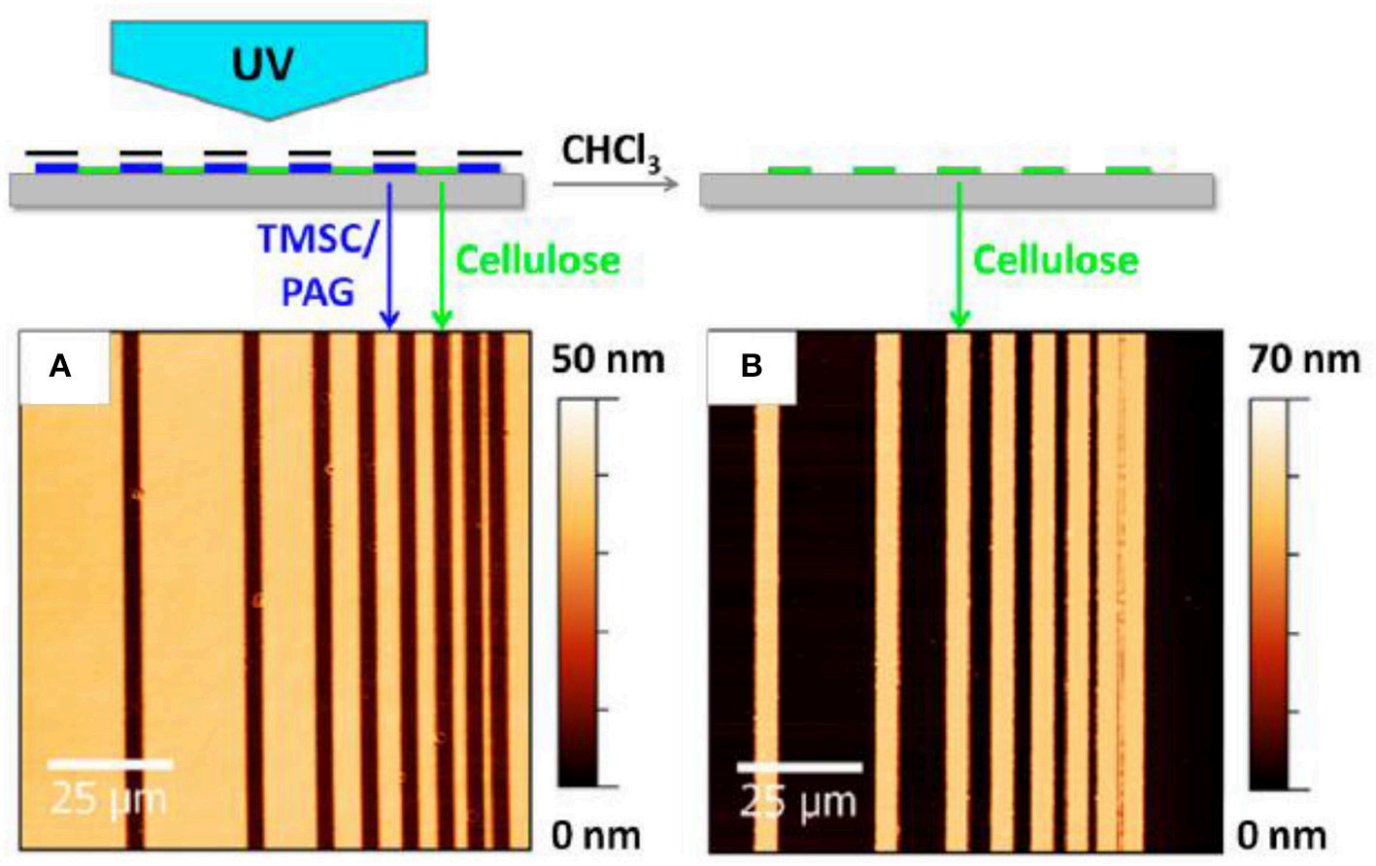
Atomic force microscope image of the cellulose thin before **(A)** and after **(B)** regeneration. Reproduced with permission from Wolfberger et al. (2014).

Sprik et al. reported a photo regeneration of cellulose film obtained from spin coating a TMSC solution onto a silica substrate (Wolfberger et al., 2014). TMSC was dissolved at 1 wt% in a chloroform solution containing 2 wt% of *n*-Hydroxynaphtalimide triflate (NHT). The formed TMSC derivative films are 60 nm thick. Upon exposing the film surface to ultra-violet (UV) (λ < 400 nm) for 10 min, the NHT produces triflic acid (CF_3_SO_3_H) which regenerates TMSC films into cellulose by substituting the trimethylsilyl group for hydroxyls. The Fourier transform infra-red (FTIR) spectra confirmed removal of the Si-O-C band. The presence of NHT does not affect the quality and properties of the cellulose film which is about 60–70 nm thick as measured by atomic force microscopy (AFM) (Figure 3). This is the finest resolution of cellulose patterns achieved.

The TMSC based cellulose films were used to make structured pattern (Mohan et al., 2013; Niegelhell et al., 2016). Rupert Kargl et al. prepared the cellulose pattering by pressing the cellulose thin film (regenerated from spin coated TMSC film) on a glass slide supporting the polydimethylsiloxane (PDMS) microchannel mold. Immediately after pressing, the microchannel were filled with the enzyme cellulase. The cellulose area exposed to cellulase was digested while the non-exposed part creates a pattern. In a second method, the TMSC film was covered with an aluminum mask with a 16 squared pattern holes. The film was exposed to HCl for regeneration into cellulose followed by cellulase exposure to remove the regenerated cellulose. Upon removing aluminum mask, the substrate surface retained the pattern of TMSC film which was finally regenerated to cellulose by acid hydrolysis (Kargl et al., 2013; Bracic et al., 2014).

**Figure 4:**
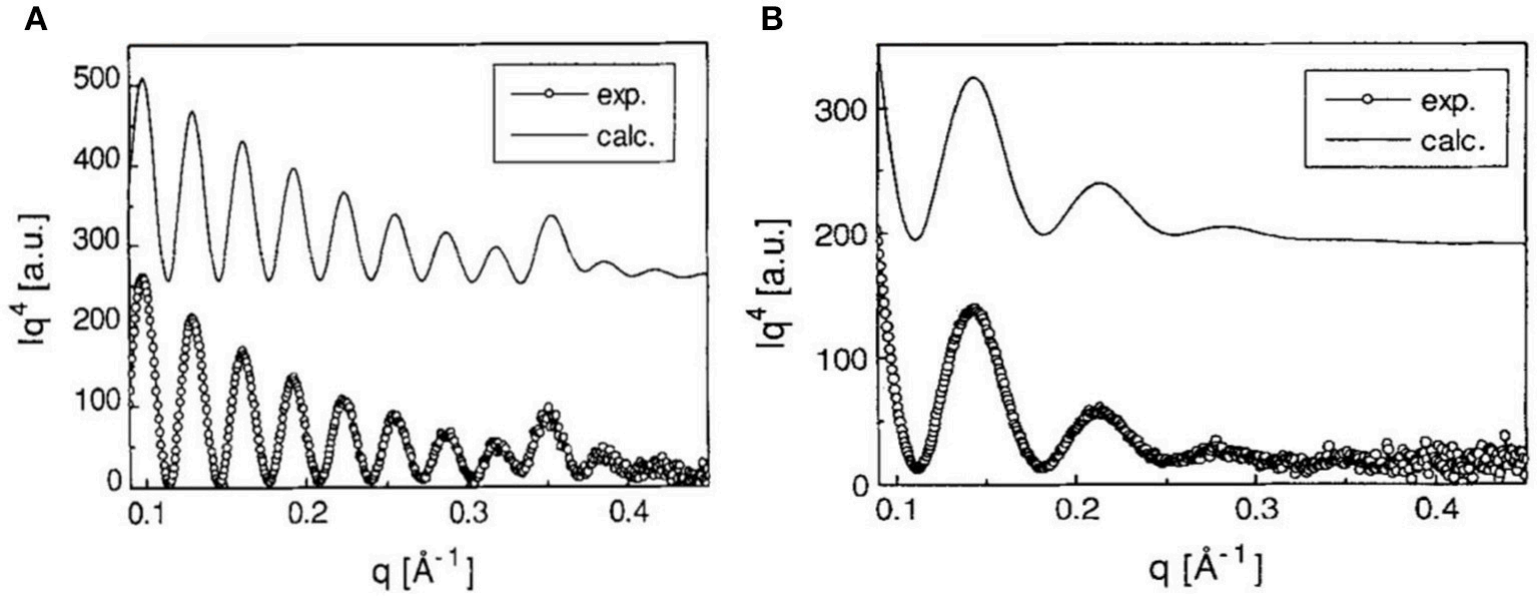
**(A)** X-ray reflectivity curves of multilayers of TMSC films at the Si substrate. **(B)** X-ray reflectivity curves are from the cellulose thin film regenerated from TMSC by HCl acid hydrolysis. Dotted are the experimental curves and solid line are the modeling curves. Reproduced with permission from Schaub et al. (1993).

Orelema et al. made cellulose films from a TMSC solution using the LS method on a polystyrene precoated gold interface (Orelma et al., 2011). The desilylation in acid vapor regenerated cellulose into films 18 nm thick and of 0.5 nm roughness with 53% crystallinity. Schaub et al. produced cellulose ultra-thin films from TMSC multilayers (Schaub et al., 1993). TMSC was prepared by reacting cellulose in dimethylacetamide/lithium chloride with hexamethyldisilazane solvent. The synthesized TMSC in *n*-hexane was spread on the water surface to form TMSC multilayers onto the silicon substrate using the LB method. X-ray reflectivity on 22 multilayers of TMSC film revealed a thickness of 19.7 nm and a roughness of 0.5 nm (Figure 4). Exposing a multilayer film to 10% HCl vapor for 30 s cleaves the TMS side groups and regenerates cellulose film 7.8 nm thick with roughness of 0.8 nm. Infra-red spectroscopy confirmed the removal of TMS side group as the characteristic Si-CH_3_, and Si-O-C vibration bands disappeared.

Cellulose thin films were also made by spin coating on a silicon substrate a solution of cellulose acetate (DS = 2) dissolved in acetone (Su et al., 2015). The acetone evaporates quickly to form a homogenous hydrophobic cellulose acetate layer on the substrate. The cellulose film was regenerated by exposing the coated cellulose acetate film to methanol-sodium methoxide (1:50) for 12 h. AFM surface analysis showed different film surface morphology before and after regeneration. AFM combined with X-ray and neutron reflectivity revealed films to be homogeneous, smooth, and 20 nm thick with a roughness <3 nm.

### Films From Native Cellulose

Cellulose thin films can be prepared by dissolving native cellulose processed from pulp and bacteria sources. Su et al. dissolved bacterial cellulose [generated from Gluconacetobacter xylinus (Römling and Galperin, 2015)] in 1-butyl-3-methylimidazolium chloride ionic liquid (Su et al., 2016). The dissolved cellulose forms silane derivatives in solution. Later, the ionic liquid was replaced with toluene. The cellulose/toluene solution produced a smooth hydrophobic film upon spin coating on a silicon substrate. Pure cellulose film is regenerated by exposing the derivative film interface to HCl vapors for 5 min which cleaves the silane derivative groups and regenerates the hydroxyl groups. Attenuated total reflectance FTIR (ATR-FTIR) confirms the substitution of all silane groups for hydroxyls (Su et al., 2016; Raghuwanshi et al., 2017b).

**Figure 5:**
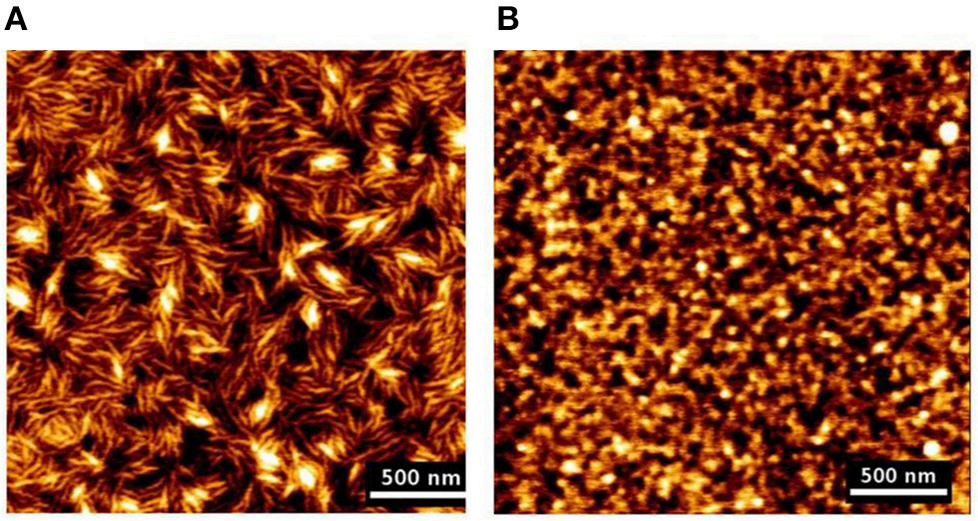
**(A)** AFM image of the deuterated bacterial cellulose (DBC) film dissolved in the ionic liquid. **(B)** AFM image of the cellulose film regenerated from the DBC in the HCl acid hydrolysis. Reproduced with permission from Su et al. (2016).

AFM and neutron reflectivity measurements indicate the formation of smooth and homogeneous cellulose films, about 10 nm thick with roughness <2 nm (Figure 5). The film swells to 2–3 times its original thickness (up to 30 nm) and revealed an absorbed water content of 70%.

Deuterated cellulose thin films of different deuteration levels were produced by dissolving deuterated bacterial cellulose (DBC). The bacterial cellulose grown in the D_2_O water environment lead to the formation of DBC, replacing all the -OH and -CH with -OD and -CD groups. The deuterating cellulose thin film interface provides significant neutron scattering length difference (SLD or contrast) between cellulose and adsorbed biomolecules. This greatly helps in visualizing and quantifying the adsorbed biomolecules at the cellulose interface (Raghuwanshi et al., 2017a).

Microcrystalline cellulose (MCC) dissolved in dimethylacetamide and lithium chloride (DMAc/LiCl) was spin coated onto silica substrates precoated with cationic polyacrylamide (CPAM) (Eriksson et al., 2005). Rinsing with the Milli-Q water and heating for 1 h at 150°C produces a cellulose film strongly adhering to the substrate. The CPAM precoating enhances the adhesion of cellulose thin film at the substrate. Ellipsometer and AFM investigations show the cellulose film to be 30 nm thick with a RMS roughness of 5 nm. The film is isotropic, and free of solvents, and structure artifacts. In the aqueous media, the thin film swells to about 3 times its original dry thickness (up to 90 nm).

Cellulose nanofibers (CNF) and cellulose nanocrystals (CNC) suspensions can be coated onto a substrate. The coated film does not require any post cellulose regeneration process. As CNF and CNC are negatively charged, a surface pre-treatment (cationic) of the substrate is required. CNF films were produced by spin coating a 0.148 wt% CNF aqueous suspension onto silica and gold substrates (Ahola et al., 2008; Orelma et al., 2012). To increase the CNF coverage, 3-aminopropyltrimethoxysilane (APTS) was used as anchoring ligand for silica while polyvinyl amine (PVAm) was selected for gold substrate. Quartz crystal microbalance (QCM) and surface plasmon resonance (SPR) results show the anchoring substances to have no effect on the CNF film properties.

CNC has a low aspect ratio (diameter 5–10 nm, length 30–100 nm) compared to CNF (diameter 30–100 nm, length 1–2 μm). Prathapan et al. spin coated a CNC layer on a uniaxially stretched polydimethylsiloxane (PDMS) precoated with poly(ethylene oxide) (PEO) (Prathapan et al., 2018). The uniaxially stretched PDMS created a surface with well-aligned grooves and low roughness. The CNC coating was transferred (wet stamped) to the poly (amino amide) epichlorohydrin (PAE)-coated glass substrate. The transferred PAE-CNC layer was heated at 105°C for 5 min which initiates covalent interactions between PAE and CNC, and creates strongly adhesion onto the glass substrate. The films formed are transparent and AFM image analysis reveals the CNC coating to be 35 nm thick layer with a RMS roughness of 5 nm. These CNC films do not swell in aqueous environments which reduces the diffusion of adsorbed biomolecules into the film structure. This technique produces patterned strips of CNC crystals 50 nm wide, 100 to 200 nm thick and of variable, and controlled periodicity ranging from 450 to 670 nm.

Eriksson et al. compared CNF and CNC films prepared by spin coating using 3 different procedures (Eriksson et al., 2007). The films showed different crystallinity ratio, morphology, and quality varying upon preparation procedure. First, CNC films were prepared by spin coating colloidal CNC suspension on a polyvinylamine (PVAm) pre-coated silicon wafers (Eriksson et al., 2007). Heat treatment at 90°C for 4 h removed the sulfur impurities from the film. Ellipsometry and XRD revealed the film to be 120 nm thick and made of cellulose I phase (Edgar and Gray, 2003; Eriksson et al., 2007).

Second, cellulose films were made by dissolving cellulose pulp in *n*-methylmorpholine *n*-oxide (NMMO), and dimethylsulfoxide (DMSO) followed by spin coating the cellulose suspension on a precoated PVAm silicon wafer. Ellipsometer showed the film to be 30 nm thick. XRD revealed the formation of a cellulose II phase with 50% crystallinity (Gunnars et al., 2002; Falt et al., 2004). In the third procedure, the cellulose film was produced by dissolving pulp in a water-methanol solution. Later, the suspension was solvent exchanged with dimethylacetamide (DMAc), and mixed with a 110°C pre-heated DMAc/LiCl suspension. The cooled clear suspension was spin coated onto the pre-coated cationic PVAm silicon wafers. The final films were obtained by washing the film surface with Milli-Q water to remove any excess LiCl. Ellipsometry showed the formation of an amorphous cellulose phase 44 nm thick (Berthold et al., 2004).

Habibi et al. created cellulose films by depositing CNC onto a thiolated gold coated substrate using the LS method (Habibi et al., 2010a). A cationic surfactant dioctadecyldimethylammonium bromide, (DODA)-Br was used as an intermediate for the adsorption/adhesion of negatively charged CNC onto the negatively charged gold substrate. In the film preparation, DODA was spread over the aqueous suspension of CNC placed in a Langmuir trough equipped with a Wilhelmy film balance. After stabilization, the surface pressure was created by reducing the distance between the trough. The DODA/gold substrate was contacted horizontally with the compressed surface to generate the CNC-DODA film.

**Figure 6:**
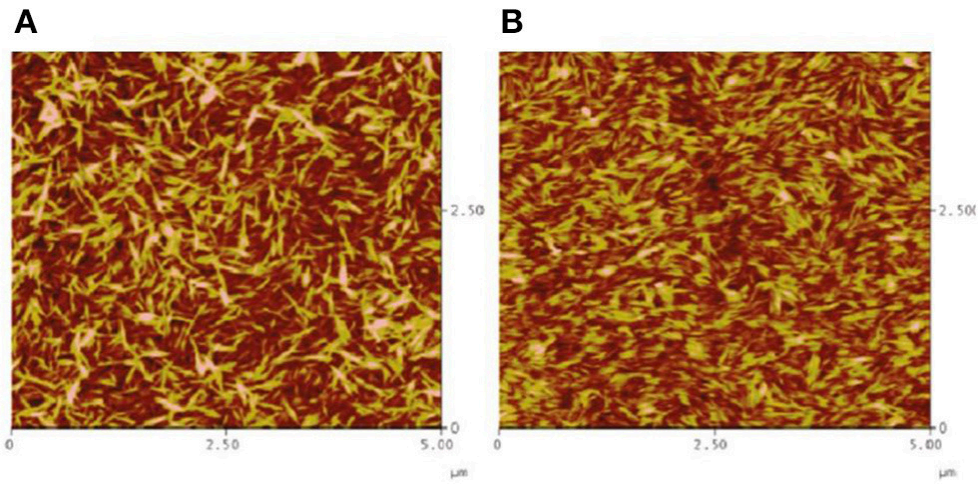
AFM height images of LS films of CNC transferred at different surface pressures, **(A)** 45 mN/m and **(B)** 60 mN/m. Reproduced with permission from Habibi et al. (2010a).

AFM and ellipsometry revealed the formation of a 10 nm thick CNC film having an average roughness of 3.7 nm. The films are stable, smooth, and strongly attached to the gold/DODA interface. Interestingly, AFM images showed that the CNC transferred to the gold/DODA at 45 mN/m pressure led the formation of an isotropic randomly distributed structure of the CNC-DODA complex. However, at even higher surface pressure (60 mN/m), the CNC-DODA complex transit into more ordered nematic anisotropic structures (Figure 6). XPS measurements of films clearly showed the carbon and oxygen peaks correspond to the pure cellulose structure. Other C-H, C-O, and sulfur XPS peaks resemble the presence of DODA and the thiol group at the gold interface. Compelling and detailed morphology quantification of cellulose phase transitions in thin films reported in literature are rare.

**Figure 7:**
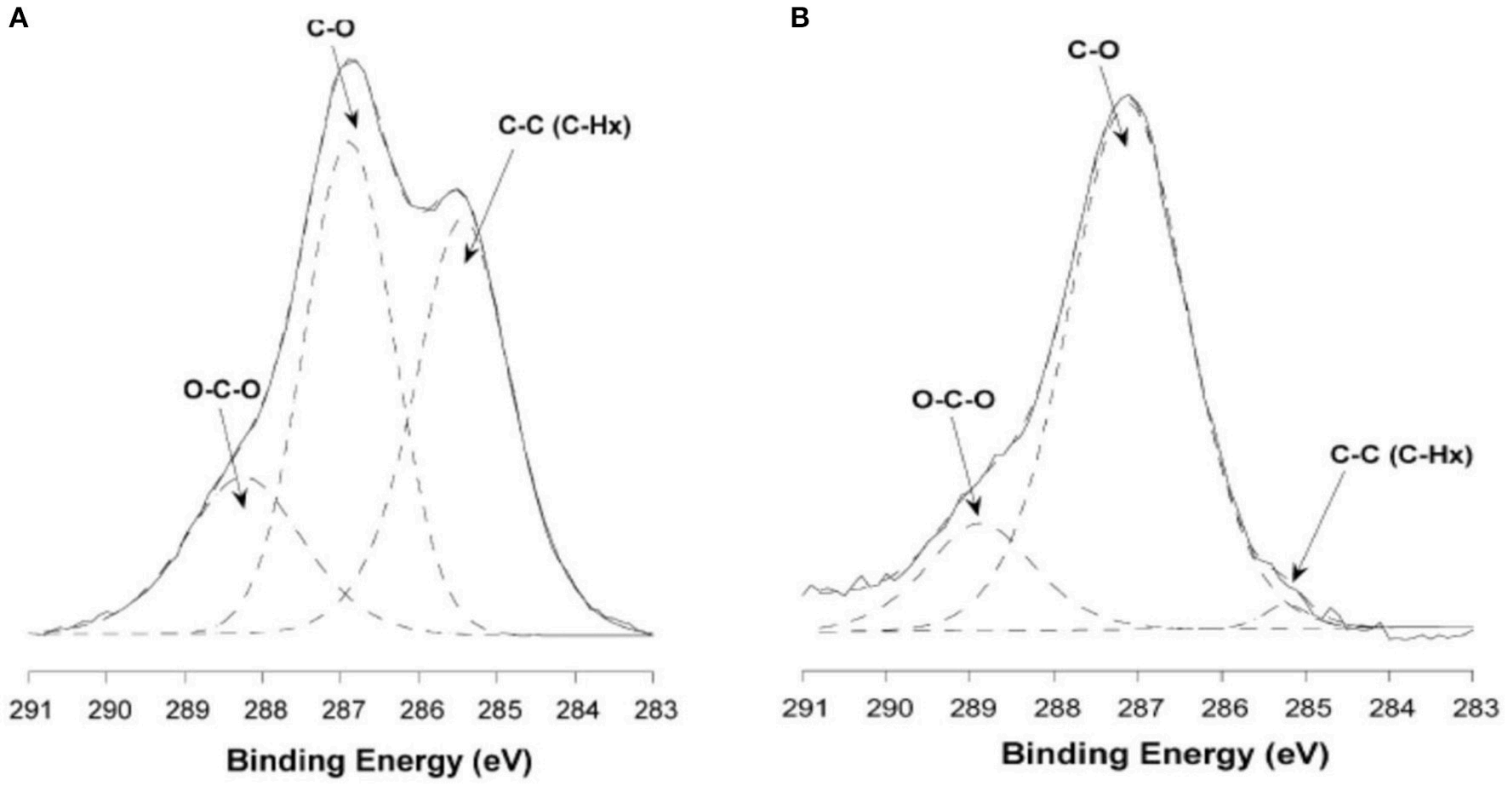
X-ray photoelectron carbon spectra of untreated **(A)** and washed ramie **(B)** cellulose nanocrystalline films. Reproduced with permission from Habibi et al. (2007).

Habibi used the same procedure to make cellulose films with the LB method (Habibi et al., 2007). In contrast to the LS films, the film interface was rinsed with chloroform, and NaOH after the film deposition to remove the adsorbed DODA and sulfur groups. Ellipsometry and AFM measurements showed the formation of a 10 nm thick CNC-DODA film before rinsing. After chloroform rinsing, the film thickness reduced to 9.2 nm indicating removal of the 0.8–1 nm thick DODA layer. The rinsed film roughness was about 3 nm. XPS investigation showed the presence of 4 kinds of bonds: C-O, C-H, C-C, and O-C-O. The C-O bonds correspond to the cellulose structure while the C-C bond (without O) corresponds to the DODA group. The XPS spectra clearly indicate the removal of any DODA traces after washing with chloroform and NaOH (Figure 7). Both LB and LS film preparation techniques produced smooth and stable thin film interfaces useful for biomolecules interactions.

## Cellulose Film Characterization

Characterization of nano scale thick cellulose films is a challenging task (Foster et al., 2018). The characterization of cellulose thin film includes quantification of composition, swelling, morphology, homogeneity, thickness, and roughness. Only a few characterization methods can measure thickness, roughness, and composition of coated films (Wenzel et al., 2005; Kassavetis et al., 2012). In many cases, the information/signals from the base substrate dominates the required characteristics from the film interface. This is due to the low volume fraction of film producing less signals compared to the dominating substrate signals.

X-ray/neutron reflectivity is a non-destructive method which determines the film thickness, roughness, and composition (Russell, 1990; Daillant and Gibaud, 2009). However, a pre-knowledge and extensive scattering curve modeling is required to extract relevant information. Swelling behavior of cellulose thin film spin coated at the silicon substrate was revealed by monitoring variations in film thickness by neutron reflectivity (Cheng et al., 2011; Raghuwanshi et al., 2017a). The cellulose film swells by 2–3 times its initial thickness of 8–10 nm. Furthermore, neutrons are sensitive to the large scattering length density (SLD) of hydrogen (H) and deuterium (D) (Shu et al., 2000; Ashkar et al., 2018). This difference can be exploited to visualize and quantify the biomolecules adsorbed at the cellulose thin film interface. Su et al. produced deuterated cellulose thin film from deuterated bacterial cellulose in which the liable –CH and -OH groups of the cellulose thin film were replaced by –CD and –OD groups. The deuterated cellulose interface provided significant SLD difference (contrast) between cellulose and adsorbed biomolecules (IgG and HRP) (Su et al., 2016). NR revealed the IgG layer to be adsorbed in the standing conformation, to have a thickness of 12.6 nm, a volume fraction of 15–20% and a surface coverage of 2.5 mg/m^2^ (Raghuwanshi et al., 2017a).

Ellipsometer is another non-destructive scattering method to measure film thickness by evaluating the refractive index of the film (Barth and Keilmann, 1993; Tompkins and Irene, 2005). Karabiyik et al. used ellipsometer to determine the refractive index (RI) of thin films of TMSC (RI: 1.46), regenerated cellulose (RI: 1.51), and cellulose nanocrystals (RI:1.51) (Karabiyik et al., 2009). The PVAm-NFC bilayer thickness on a PDMS substrate was measured to be 2.2 nm and the refractive index in the range 1.56–1.52 was measured by variable angle (50–70°) spectroscopic ellipsometer. The layer was modeled with the Cauchy model (Eita et al., 2011).

**Figure 8:**
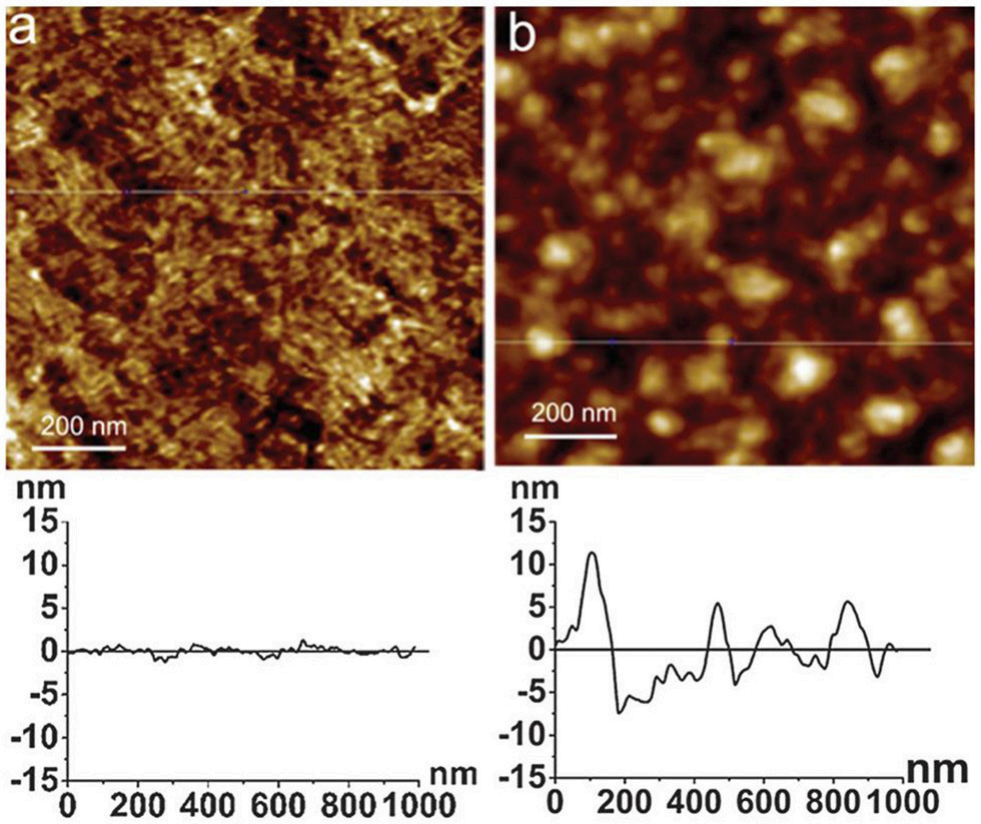
AFM images and the corresponding surface profiles for **(a)** mostly crystalline cellulose film, **(b)** amorphous cellulose film on polystyrene coated gold substrates. The scan size is 1 mm^2^ and the z-range is 15 nm. Reproduced with permission from Tammelin et al. (2015).

Atomic force microscopy (AFM) is a powerful method to quantify surface morphology, film thickness, and roughness of cellulose nanoscale thin films (Dufrêne, 2002; Lahiji et al., 2010). A cantilever is scanned continuously or tapped over the film surface; it measures surface forces in nano-newtons, and provides film surface topography within the resolution of 0.2 nm. Notley et al. used AFM to measure surface forces between cellulose thin films (120 nm thickness), and amorphous cellulose spheres (Notley and Wagberg, 2005; Notley et al., 2006). Tina Maver characterized regenerated cellulose thin film produced by TMSC on a silicon substrate. AFM image analysis revealed the TMSC spin coated film is 54 nm thick; after regeneration by acid, the film thickness decreased to 19 nm (Maver et al., 2015). Tammelin et al. used AFM to differentiate the surface morphology of the cellulose thin films (thickness 33 nm) prepared by LS and SC methods (Tammelin et al., 2015). The LS method produces highly crystalline film with a RMS roughness of 0.5 nm while the SC method generates amorphous film with RMS roughness of 3.4 nm. The LS films are much smoother than the SC films which is clearly seen in the AFM images (Figure 8).

Fourier transform infra-red (FTIR) spectroscopy is a convenient technique to determine the different chemical functionalities present in the cellulose thin film by adsorption of IR radiation (Müller and Schmitt, 1997). For characterizing thin films of nanometer thickness, attenuated total reflectance FTIR (ATR-FTIR) is used. FTIR spectra indicate chemical bonds, functional groups and help in identifying organic molecular groups and compounds, side chains and cross links which correspond to the characteristic vibrational frequencies in the IR range.

**Figure 9:**
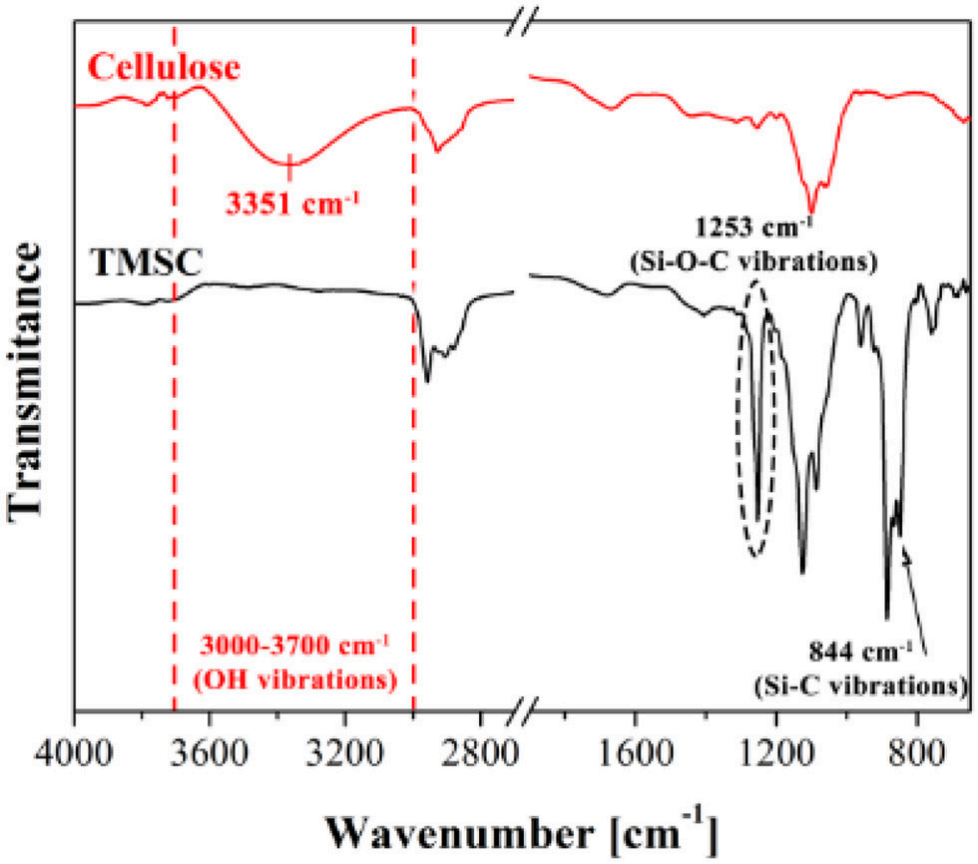
ATR-FTIR spectra of cellulose and TMSC films showing different vibrational bonds. Reproduced with permission from Maver et al. (2015).

ATR-FTIR investigation is used to track and confirm the generation process of TMSC films into cellulose film upon acid hydrolysis. Figure 9 shows TMSC film before and after regeneration (Maver et al., 2015). TMSC film spectra show C-Si vibration at 844 and 1,253 cm^−1^. In the regeneration process, the Si-C bond disappears confirming the removal of Si-C groups. Moreover, the appearance of –OH bonds at 3,351 cm^−1^ combined with other peaks (between 1,200 and 1,000 cm^−1^) reveal a cellulose film. Su et al. performed ATR-FTIR on the deuterated cellulose thin film to confirm the replacement of the –CH groups of cellulose chain by –CD groups. ATR-FTIR showed the C-H stretching at 2,900 cm^−1^ from the hydrogenated cellulose is shifted to 2,100 cm^−1^ which corresponds to the C-D stretching (Su et al., 2016).

Quartz crystal microbalance with dissipation (QCM-D) is a surface sensitive method to measure the variation in the cellulose thin film morphology and its interaction with the biomolecules in nanograms and in real time (Marx, 2003). The dissipation mode of the QCM-D reveals the rigidity of the adsorbed biomolecules layer at an interface (Turon et al., 2008). Raghuwanshi et al. characterized the adsorbed IgG layer at the cellulose thin film interface. They measured the adsorbed IgG layer to be 140 nm thick with a volume fraction of 25% and a surface coverage of 4 mg/m^2^ (Raghuwanshi et al., 2017a).

**Figure 10:**
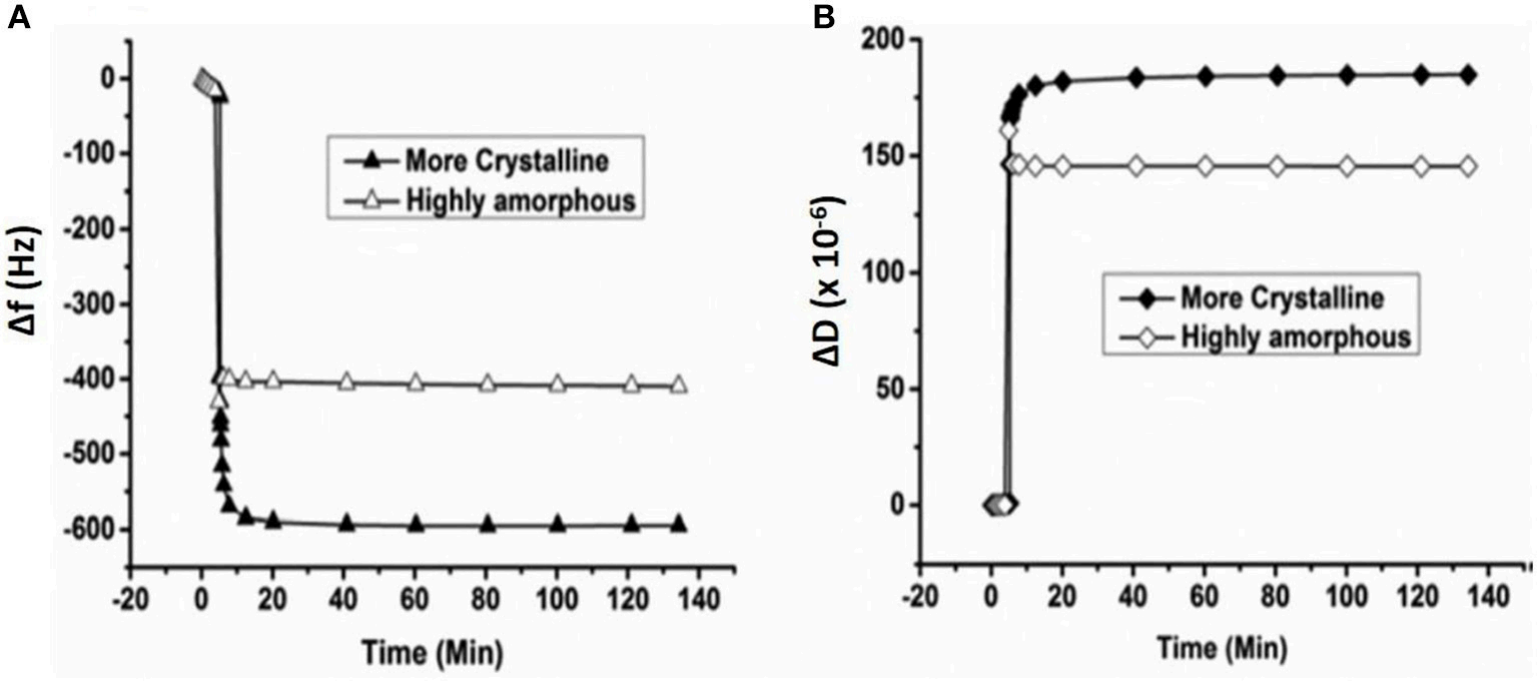
QCM-D measurement of the water absorption in amorphous and crystalline cellulose using thin films. Change in frequency **(A)** and change in dissipation **(B)** as a function of time upon exposure to water for more crystalline and highly amorphous cellulose films. (f_0_ = 5 MHz, *n* = 3, f_3_/n). Reproduced with permission from Tammelin et al. (2015).

Enzymatic digestion of cellulose thin film 24.2 nm thick was also monitored by QCM-D. Investigation showed the loss of 117 ng/cm^2^ of cellulose from the sensor surface after 15 h of enzymatic treatment (Cheng et al., 2011). Kittle relied on QCM-D to investigate the equilibrium water content of cellulose films by solvent exchange (Kittle et al., 2011). QCM-D experiments were performed to compare the water content in films of regenerated cellulose (RC), sulfonated nanocrystalline cellulose (SNC), and desulfonated nanocrystalline cellulose (DNC). QCM-D showed the RC exhibit 5 water molecules per anhydroglucose unit, while both SNC, and DNC retained five time more water in the films. Tammelin et al. relied on QCM-D to characterize the swelling and water uptake/release behavior of amorphous and crystalline cellulose thin films (33 nm thick). QCM-D showed the crystalline films absorb more water than the amorphous cellulose film (Figure 10), where the mass change (ΔF) in the crystalline film are higher than for the amorphous film. The degree of swelling for the crystalline film is 72% compared to 4.8% for the amorphous film. QCM-D results indicate that the crystalline films have a nano-porosity structure and a higher surface area than the amorphous film which bind a large amount of water molecules (Tammelin et al., 2015).

X-ray photoelectron spectroscopy (XPS) is a quantitative method to determine the surface composition and impurities of the cellulose thin film interface (Johansson and Campbell, 2004). In XPS, the bombarded X-rays photons eject core, and valence band electrons from the film surface which are analyzed for the composition of the film (Swartz, 1973). XPS determined the composition of cellulose thin films prepared by dissolving microcrystalline cellulose in 50%wt water/*n*-methylmorpholine-n-Oxide (NMMO) at 115°C, and then coated onto silica and gold substrates. The cellulose film is anchored by polyvinylamine polymer. XPS spectra shows no peak from the nitrogen (400 eV) of NMMO which reveals the cellulose film to be solvent free. The carbon emission spectra of the XPS spectra shows the significant contribution from the bonded carbon atoms (O/C ratio is 0.85; O-C-O/C-O ratio is 0.22) in the cellulose film structure (Song et al., 2009).

**Figure 11:**
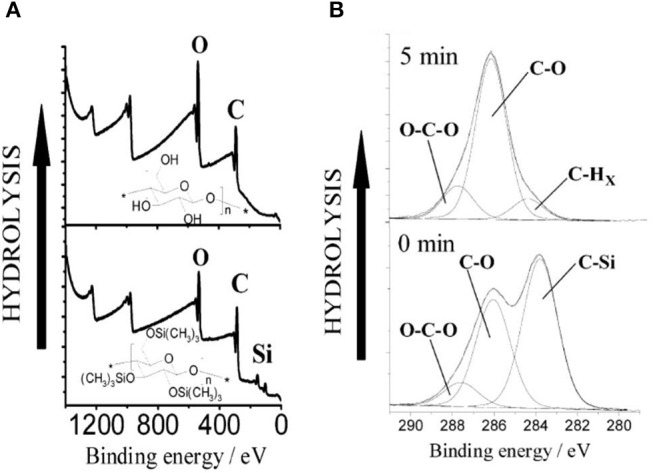
**(A)** XPS wide scans from trimethylsilylcellulose (TMSC) and cellulose films hydrolyzed from TMSC with 2 M HCl for 1 min. **(B)** Hydrolysis of TMSC films with 0.5 M HCl followed with XPS. The carbon emission is resolved to contributions illustrating the decline of silicon bonded carbon from TMSC, as the hydrolysis proceeds. Reproduced with permission from Kontturi et al. (2003).

Kontturi studied the hydrolysis of trimethylsilyl cellulose (TMSC) and its conversion in pure cellulose film by XPS (Kontturi et al., 2003). XPS survey spectra show the removal of the Si group upon the hydrolysis of TMSC film which reveals the cleaving of –SiO bonds (Figure 11). The detailed XPS carbon spectra of the TMSC film show the presence of O-C and O-C-O and C-Si bonds coming from the TMSC structure. The XPS carbon spectra after 5 min of HCl hydrolysis reveal the complete removal of the C-Si bond; and the O-C and O-C-O bonds corresponding to the cellulose remains.

## Cellulose Thin Film: Surface Functionalization and Biomolecules Interactions

Engineering the specific and non-specific adsorption of biomolecules at the cellulosic film interface is critical in designing effective and biodegradable diagnostics and low-cost sensors. The negative surface charge of cellulose, with its 3 liable -OH groups (per monomer), can be used to modify the interface for specific biomolecules interaction, and functionality. Biomolecules interact with the cellulose film by forming physical and chemical bonds. The physical bonds are due to the van der Waal, ionic, and electrostatic interactions, and the chemical bonds are by covalent interactions.

Cellulose thin film of large surface/volume ratio, low porosity and able to form hydrogen bonds at the interface can overcome many challenges of bulk cellulose materials such as paper and textile. The limiting issues with bulk cellulose interacting with nanoscale biomolecules are their high water absorption, high roughness, and high porosity. The structure of paper and textile is also complex and presents heterogeneous morphologies. This means a high fraction of biomolecules disappears by absorption into the bulk of cellulose. Also, the biomolecules adsorb on the external surface into a plethora of conformations which can causes an important distribution of available functionalities and interaction with the molecules probed by paper/threads diagnostics. The low porosity of nanoscale thin films hinders the diffusion of biomolecules within the film structure as they remain at the interface for their specific functionality.

The essential requirements and characteristics of effective cellulose thin films to interact with nano sized biomolecules are:

- Controllable thickness in the nanometre range (1–200 nm) for films of large surface area.
- Smooth film interface of roughness lower than the biomolecule critical dimension (< 1 nm) for accurate size measurement and ensuring homogeneous biomolecules conformation.
- Low porosity for limited water intake capacity (if hydrophobic) for increased biomolecules longevity at the interface.
- An interface which is easy to functionalize to adsorb large amounts of specific biomolecules for selective activity with target molecules.
- Transparent and controllable wettability.

Mohan et al. studied the unspecific bovine serum albumin (BSA) adsorption at the cellulose interface with films modified with cationic cellulose derivatives: cellulose 4-([*nnn*-trimethylammonium] butyrate chlorides) with different degree of substitution (DS) of NMe^3+^Cl^−^ (Mohan et al., 2014). QCM-D (wet mass) combined with MP-SPR (dry mass) show 2 times higher adsorption of fluorescently labeled BSA molecules on the modified cellulose interface (BSA wet mass: 42 mg/m^2^, Dry Mass: 3.9 mg/m^2^) compared to the pure cellulose (BSA wet mass: 20 mg/m^2^, Dry mass:1.6 mg/m^2^) at pH 5.0. Higher BSA adsorption indicates higher amount of positive surface charges induced by the cationic cellulose derivative at the cellulose interface. However, the non-fluorescently labeled BSA molecules might show different mass adsorption and conformation at variable pH. The fluorescent labeling can alter the native conformation of BSA at a particular pH and result in a large BSA adsorption at an interface.

Eriksson et al. studied the degradation of 30 nm thick cellulose film exposed to active and inactive enzymes (Eriksson et al., 2005). Cellulases were adsorbed directly at the cellulose interface without any surface pre-modification. An inactive enzyme D10N was adsorbed at 3 mg/m^2^, forming an enzyme monolayer 2.5 nm thick. However, no film degradation was observed by inactive enzymes as the mass over the substrate remained unchanged. In contrast, exposing the film surface to an active cellulase (GH45) embedded with carbohydrate-binding module (CBM) simultaneously starts adsorbing onto and degrading the film interface. The overall film mass first increases due to enzyme adsorption and later decreases as cellulose degrades. High enzyme concentration leads to large adsorption and faster film degradation. In the absence of CBM, no adsorption of active enzymes was observed on the film interface and no cellulose degradation occurred.

**Figure 12:**
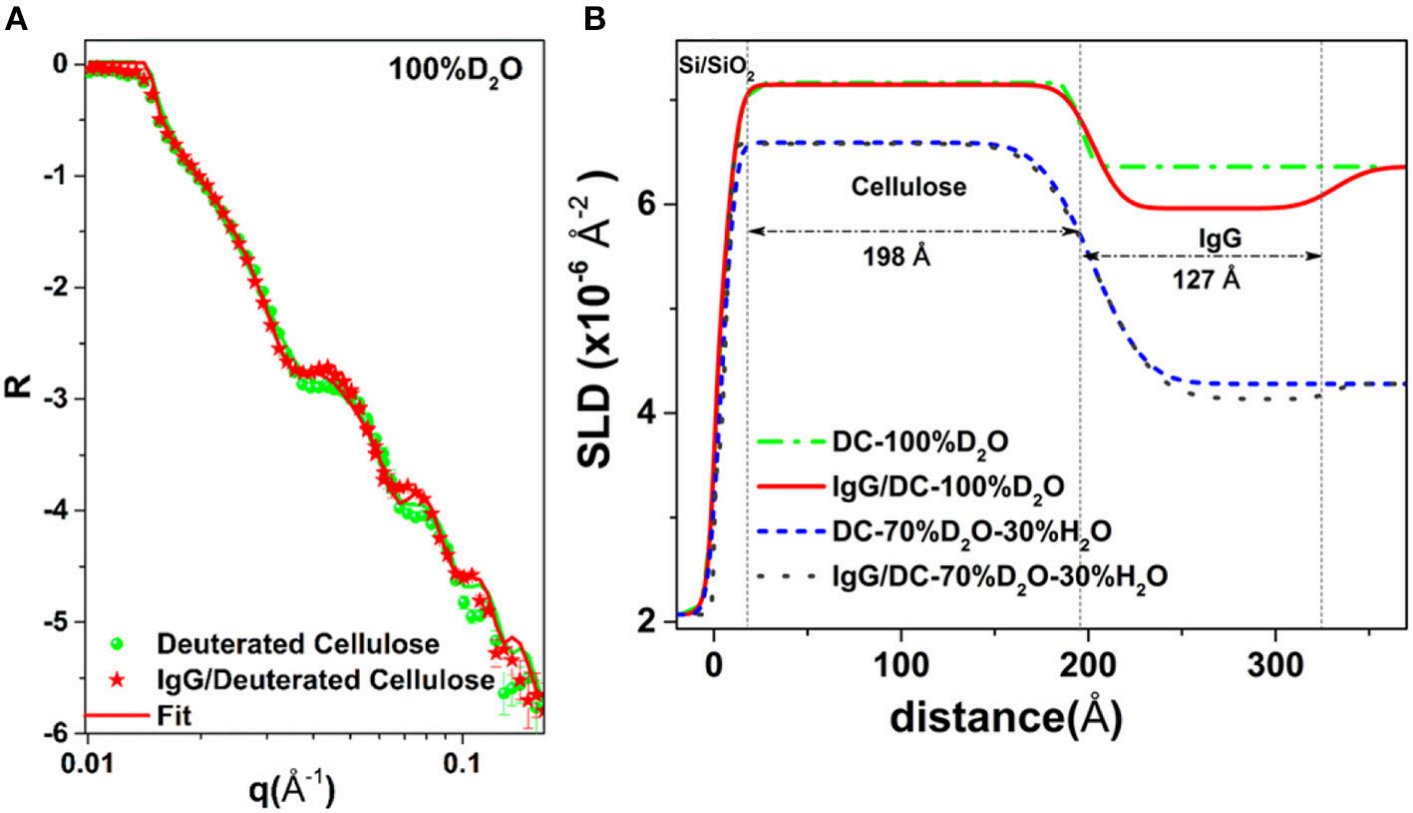
**(A)** NR curves of deuterated cellulose and IgG adsorbed deuterated cellulose. **(B)** SLD profile with thickness from the substrate interface obtained by fitting the NR curves. Reproduced with permission from Raghuwanshi et al. (2017a).

Raghuwanshi et al. adsorbed two types of biomolecules: an immunoglobulin G (IgG, antibody) and horseradish peroxidase (HRP, enzyme) onto deuterated cellulose films to create air-solid and liquid-solid interfaces. Deuterated cellulose films had all the hydrogen groups of the anhydroglucose replaced by deuterium (Raghuwanshi et al., 2017b). The replaced deuterium induces a large neutron SLD difference between the cellulose and biomolecules, providing high contrast by neutron reflectometry (NR) or scattering. In D_2_O, the SLD of deuterated cellulose (7.1 × 10^−6^ Å^−2^) and IgG (4.1 × 10^−6^ Å^−2^) clearly show the differences in the NR curves (Figure 12). The SLD of hydrogenated cellulose is 3.56 × 10^−6^ Å^−2^, which is close to the SLD of IgG and HRP (3.9 × 10^−6^ Å^−2^), and does not provide sufficient contrast to differentiate cellulose from proteins. The IgG and HRP biomolecules might also contain a variable number of liable hydrogens in their structure. Dispersion of biomolecules in D_2_O replaces the liable hydrogens by deuterium which alters their SLD. Determining the deuteration level of biomolecules can further increase accuracy in the quantification of adsorbed biomolecules in neutron-based experiments.

NR combined with QCM-D quantifies the conformation of the IgG molecules adsorbed at the cellulose interface as a monolayer of thickness 12.7 nm, surface coverage of 4.2 mg/m^2^ and forming 25% of the interphase volume fraction. The HRP molecules also adsorbed as a monolayer of thickness 9.8 nm with volume fraction of 20%; this corresponds to partial surface coverage of HRP.

A water resistant nanofibrillated cellulose thin film was functionalized by carboxylated groups using the TEMPO oxidation method (Isogai and Kato, 1998). This converts the –OH groups of the cellulose C6 hydroxyl into a -COOH functionality. Conductometric titration indicates −72 μeq/g of charge on the pure NFC film, which increases by a factor 3, up to −237 μeg/g after 5 min TEMPO oxidation (Orelma et al., 2012).

**Figure 13:**
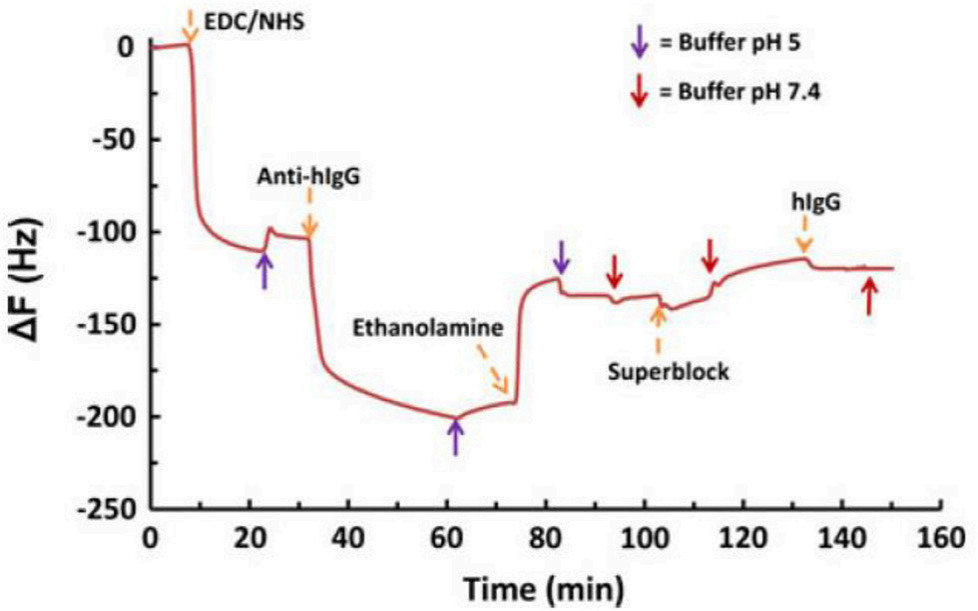
QCM-D data for the adsorption of human IgG on EDC/NHS activated NFC-film in the presence of conjugated antihuman IgG. Conjugation of IgG resulted in a small shift in frequency. Reproduced with permission from Orelma et al. (2012).

The –COOH groups of the film interface were further modified into amine reactive sites by EDC/NHS coupling reaction for BSA and IgG adsorption. QCM-D shows the oxidation increases the film water intake capacity with a film wet mass of about 25 mg/m^2^ (Figure 13). Modification of surface charge with NHS-ester reduces the water intake and produced no desorption of NHS-esters. The amount of water in the film structure can significantly affect the conformation and adsorption of biomolecules at the cellulosic interface. Therefore, quantification of water within the film structure, before and after adsorption of biomolecules, is an important factor.

The BSA adsorbed at the unmodified film interface had a surface coverage of 1.6 mg/m^2^. The adsorbed BSA molecules show irreversible binding upon rinsing with 10 mM NaCl at pH 10. In contrast, the oxidized film interface reversibly adsorbed BSA at 6.8 mg/m^2^; rinsing with 10 mM NaCl at pH 10 desorbed all BSA molecules. However, an initial BSA adsorption of 9.8 mg/m^2^ was achieved onto the EDC/NHS modified film surface. Upon rinsing, the unbounded BSA molecules were removed and the only irreversible bounded BSA (4.9 mg/m^2^) remained at the film interface.

Fluorescence imaging was used to track the adsorption of FITC-stained anti human IgG molecules on unmodified films. It shows a weak fluorescence intensity of 1.8. Rinsing with NaCl completely removed the IgG from the unmodified CNF interface. However, the EDC/NHS modified film interface showed a fluorescence 10 times stronger with an intensity of 18.76. NaCl rinsing had no effect on the adsorbed IgG as the molecules remained at the film interface with a surface coverage of 18 mg/m^2^.

**Figure 14:**
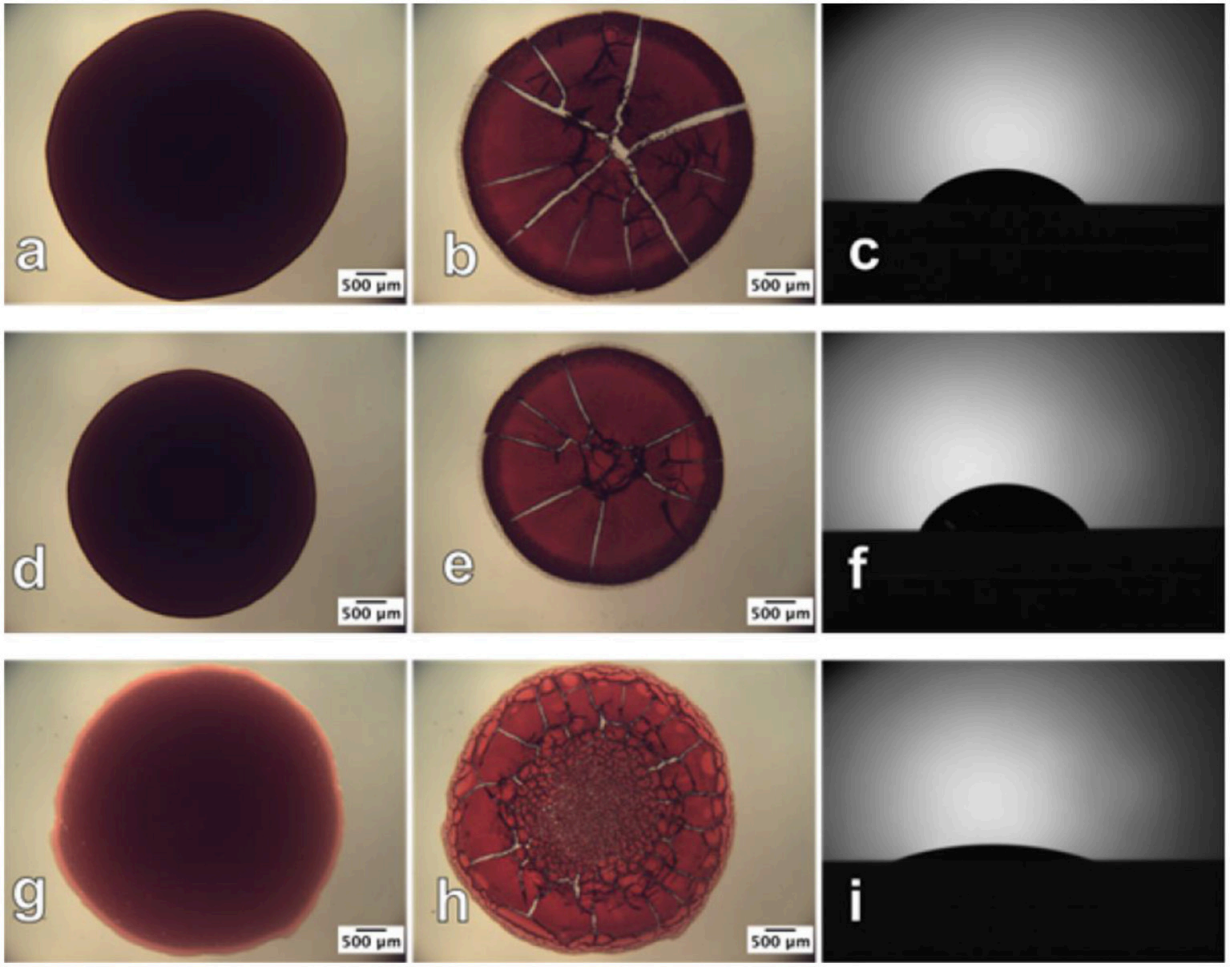
Optical images of human blood before (left) and after (middle) drying and the contact angle sessile drop image (right), respectively, on the **(a–c)** glass slide surface; **(d–f)** cellulose acetate surface; **(g–i)** regenerated cellulose surface. The before and after drying images were taken in transmittance mode. Reproduced with permission from Prathapan et al. (2018).

Prathapan et al. studied the drying profile of blood stains on CNC films (35 nm thick) coated onto a glass substrate. AFM and contact angle measurements quantified the roughness and wettability of the dried blood stains onto the CNC interface (Figure 14). Blood antibody and antigen tests proved the CNC layer capability to provide high selectivity for blood testing diagnostics (Prathapan et al., 2018).

Comparison of a CNC film with a cellulose film regenerated from cellulose acetate (Su et al., 2016) reveals significantly less swelling for CNC due to its crystalline structure. The regenerated cellulose is amorphous and swells up to 2–3 times in water. Biomolecules are prompt to diffuse into the swollen layer forming a gel or easily detach from the interface. The non-swelling CNC coating improves visualization of the blood stain reacting with an antibody during typing testing. Post hydrophobic chemical modifications of the cellulose film interface can hinder water penetration in the film structure and reduce its swelling, which can benefit further in visualization and quantification of biomolecules at film interface.

Huang et al. tested the functionality and longevity of physically adsorbed IgG and IgM antibodies on cellulose acetate films and compared those with regenerated cellulose thin films on glass substrate (Huang et al., 2017). X-ray and neutron reflectivity revealed the cellulose films to be 20.8 nm thick and swells to twice of their original thickness in water.

The adsorbed IgG and IGM antibodies were dried on cellulose acetate and regenerated cellulose film interfaces. The effect of aging on antibodies functionality was monitored by measuring the red blood cell (RBC) size agglutinates using image analysis for 30 days. Antibody aging was directly related to surface hydrophobicity by recording water droplet contact angle over the surface of antibody dried and aged onto the cellulosic films. The study revealed that both IgG, and IgM survive longer on the cellulose acetate film than on a cellulose film. This increased biomolecule longevity is due to a more favorable molecular conformation of the adsorbed antibodies as the hydrophobic cellulose acetate restricts hydrogen bonding interactions between antibody and surface which retain the original conformation of the native antibody (Huang et al., 2017).

However, IgM loses its functionality faster than IgG at both cellulosic interfaces, which might be due to the higher carboxyl content of IgM. The drop-in antibody functionality during aging is explained by the formation of antibody-surface hydrogen bonds which alter the biomolecule conformation and also rotate hydrophobic groups toward the air interface. The increased hydrophobicity of the antibody layer also delays wetting and rehydration by blood and buffers, slowing down the response of the test for analysis.

**Figure 15:**
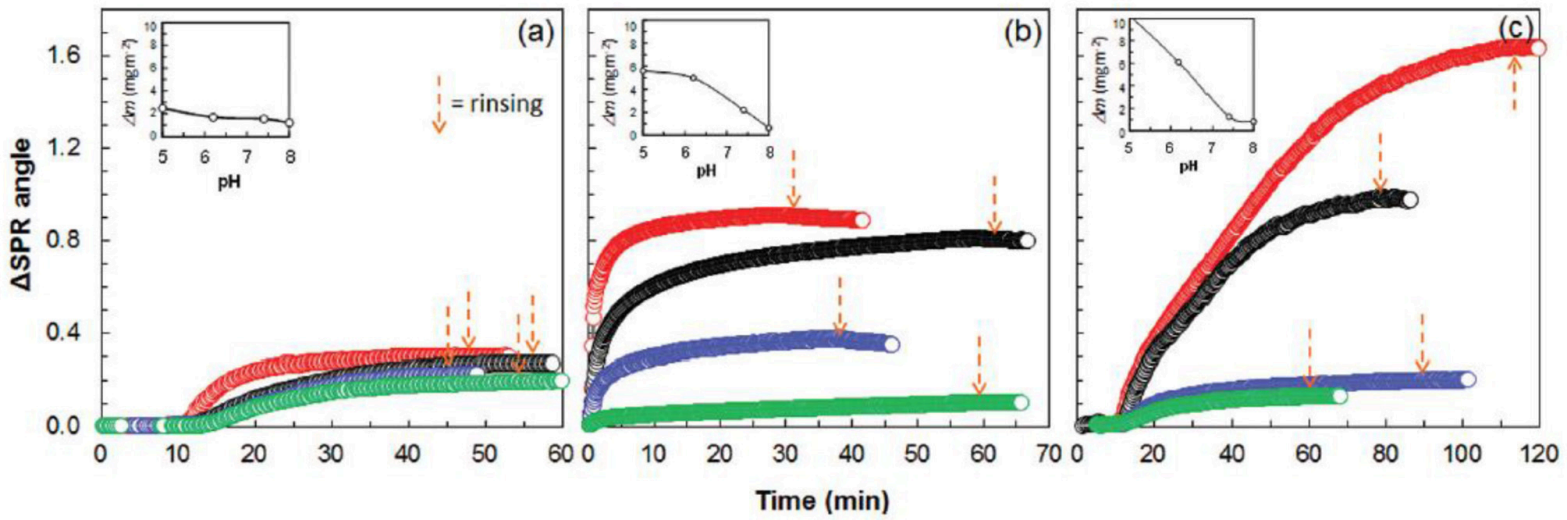
SPR sensogram on the adsorption of 0.1 mg/mL human IgG on cellulose. **(a)** CMC-modified cellulose **(b)**, and chitosan modified cellulose **(c)**, from aqueous solutions of pH 5.0, 6.2, 7.4, and 8.0 (the respective curves follow the same order from top down). Corresponding calculated adsorbed mass is indicated as a function of pH in the inserts (calculated from SPR data modeling). Reproduced with permission from Orelma et al. (2011).

The human immunoglobulin G (h-IgG) and BSA interaction with chitosan and carboxymethyl cellulose (CMC) modified cellulose interfaces were studied by QCM-D combined with SPR measurements (Orelma et al., 2011). Both h-IgG and BSA adsorbed onto the modified cellulose interfaces (Figure 15).

CMC and chitosan adsorbed on cellulose at 53 mg/m^2^ and 4.4 mg/m^2^ (wet mass), respectively. The dry mass evaluated for the adsorbed CMC was 2 mg/m^2^ and 0.7 mg/m^2^ for chitosan. The CMC modified cellulose interface adsorbed 97% of water on hydration, which is higher than the chitosan modified surface (83%). Near the isoelectric point (pH 5.0), BSA adsorption at the unmodified cellulose interface is 1.7 mg/m^2^, which increases to 3.5 mg/m^2^ on the chitosan modified interface. However, BSA adsorption does not change much at the CMC modified interface as compared to the unmodified interface.

Similarly, at pH 5.0, h-IgG also showed enhanced adsorption at the chitosan modified interface (about 10 mg/m^2^) as compared to the CMC modified (about 5 mg/m^2^) and unmodified cellulose (2.2 mg/m^2^). At higher pH 7.4, the adsorption of both BSA and h-IgG decreases for all the interfaces studied. The surface modification induces surface charges at the cellulose interface which play an important role in controlling the adsorption of biomolecules due to varying electrostatic interaction at the different pH values.

Multiple factors can affect the biomolecules interactions, longevity and functionality at the cellulosic thin film interface. These include the cellulose thin film morphology, its water intake capacity and swelling behavior, substrate types, cellulose surface chemistry/modification, surrounding pH and ionic strength, and initial biomolecules conformation and its ability to interact with the cellulosic thin film interface.

## Summary and Perspectives

The review aims at engineering biodegradable and cost effective cellulosic thin films or interfaces of controlled properties. The applications of interest range from measuring macromolecule interactions onto cellulose and quantifying biomolecule conformation at interfaces to optimizing biomedical applications. Native cellulose from different sources or cellulose derivatives can be used to form cellulose thin film by the spin coating, Langmuir-Schaefer and Langmuir Blodgett coating methods. Cellulose films can be coated directly onto a substrate or indirectly using chemical pre-modification. For measuring biomolecules interactions, the coated cellulose thin film surface requires film thickness between 2 and 100 nm and having a roughness of about 1 nm. This is to fulfill the requirements of advanced characterization surfaces technique (thickness) while being able to distinguish biomolecules from the film roughness. Smooth and ultra-thin films enable to accurately modify the cellulose surface and to increase surface area for optimizing biomolecules activity at interfaces. Cellulose surfaces can be easily functionalized to selectivity retain or increase the activity of specific bio-molecules. This enables cellulose thin films to be optimized for biomedical applications, such as blood typing, pregnancy test and adhesion of specific cell at the interface.

The current review encourages engineering smooth and ultra-thin cellulosic interfaces to specific applications. These interfaces are low cost and enable multiple functionality by reacting the cellulose hydroxyls into carboxylic or amine groups. Currently, only a few applications using biomolecules at cellulose thin film interfaces have been developed. Many questions and challenges remain to reproducibly prepare nanocellulose film and best control their interactions with biomolecules. A limiting step has long been the ability to prepare smooth cellulose film from the pre-functionalized CNF/CNC. Advance characterization can further be developed to visualize and quantify the adsorbed biomolecules at the film interface. This will enable to control the biomolecules adsorption at the cellulosic interface and their conformational changes at any pH, ionic strength and temperature required.

## Author Contributions

VR and GG both wrote the manuscript and equally contributed.

### Conflict of Interest Statement

The authors declare that the research was conducted in the absence of any commercial or financial relationships that could be construed as a potential conflict of interest.
